# The survival of a free flap after post-operative cardiac arrest and cardiopulmonary resuscitation in an 85-year-old female: A case report and literature review

**DOI:** 10.1016/j.jpra.2026.05.036

**Published:** 2026-05-30

**Authors:** Mitsutoshi Okuda, Tomoaki Kuroki, Fuko Iwai, Eri Anzaki, Takuji Yokoyama, Koshiro Tanaka, Nobuyuki Mitsukawa

**Affiliations:** a Department of Plastic and Reconstructive Surgery, Kochi Medical School Hospital, Kochi, Japan; b Department of Plastic, Reconstructive and Aesthetic Surgery, Chiba University Hospital, Chiba, Japan

**Keywords:** Free flap, Cardiac arrest, Cardiopulmonary resuscitation, Vasopressor

## Abstract

Cardiac arrest rarely occurs during or after free flap surgery, and although multiple studies have reported the outcomes of in-hospital cardiac arrest, studies describing the outcomes of free flaps in patients who were successfully resuscitated after cardiac arrest are rare. We present herein the case of an 85-year-old woman who underwent an excisional biopsy after presenting to our dermatology department with a rapidly growing subcutaneous mass in her left forearm. A pathological examination of the biopsy specimen revealed the mass to be a malignant tumour; therefore, the patient subsequently underwent an extended excision, resulting in a 100 × 250 mm defect in the dorsum of her left forearm. The defect was successfully reconstructed using a free anterolateral thigh flap from the right leg; however, 2 days post-reconstruction, the patient experienced cardiopulmonary arrest. Spontaneous circulation was returned after 10 min of cardiopulmonary resuscitation, during which 2 mg of adrenaline was administered. The free flap was closely observed for >30 days post-arrest and showed no signs of necrosis or infection during that time. The patient was transferred to a nursing facility on day 53 post-arrest owing to the loss of high cognitive function. Our institution has encountered two other cases of post-operative cardiac arrest after free flap transfer in the past 8 years, and a literature search of PubMed returned three documented cases of free flap survival after cardiac arrest and resuscitation. This case report and literature review aimed to demonstrate the characteristics of the free flaps that survived cardiac arrest and subsequent resuscitation. An analysis of these six aforementioned cases of free flaps that survived cardiac arrest demonstrated that post-operative cardiac arrest and cardiopulmonary resuscitation may not significantly decrease the survival of free flaps.

## Introduction

Post-operative cardiac arrest is a rare and potentially fatal complication of free flap surgery that has a severe impact on patient outcomes. The incidence of post-operative cardiac arrest after free flap surgery is 0.4%, based on an analysis of 22,839 cases of free tissue transfer by Diaddigo et al.^1^ Although the occurrence of post-operative cardiac arrest is rare, once a patient enters a state of cardiac arrest, their chance of survival is low. Okubo et al.^2^ analysed 348,996 cases of in-hospital cardiac arrest in the United States and reported that 22.6% of these patients survived until discharge. The probability of survival after 1 min of cardiopulmonary resuscitation (CPR) was estimated to be 22%, dropping to 9.5% after 10 min.^2^ Although multiple studies have been described the outcomes of patients after in-hospital cardiac arrest and subsequent resuscitation, reports on the survival of free flaps after cardiac arrest are limited. We report herein the case of an 85-year-old woman who was found in a state of cardiopulmonary arrest after 2 days of a free flap transfer. The patient was successfully resuscitated after 10 min of CPR, and the free flap showed no sign of necrosis or vascular compromise for at least 30 days post-cardiac arrest. We also analysed six cases of free flaps in patients who survived cardiac arrest and subsequent resuscitation and reported our findings herein.

## Case presentation

**Fig. 1:**
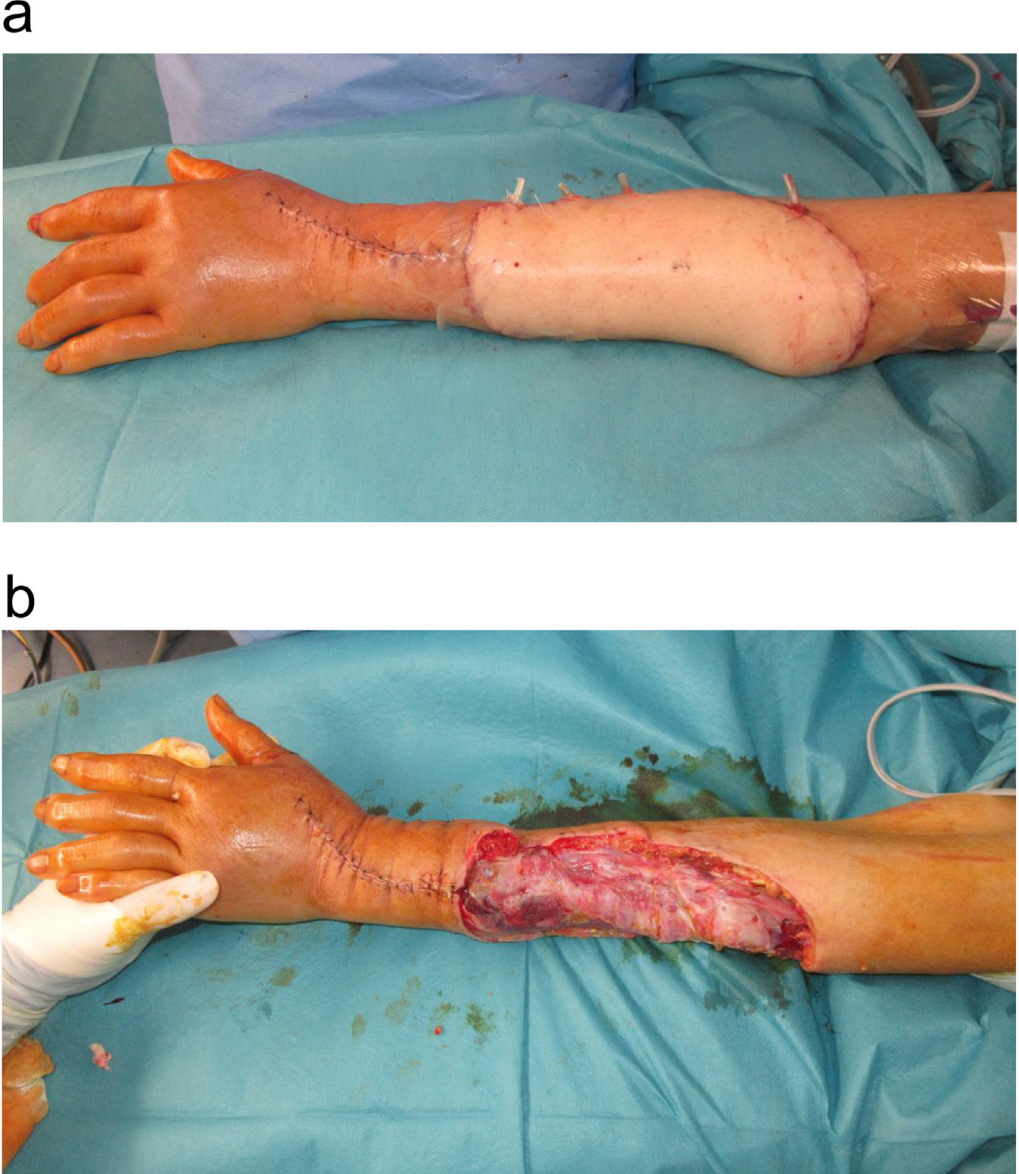
(a) Post-excision: the orthopaedic surgery team performed an extended excision that resulted in a 100 × 250 mm defect in the dorsal left forearm. (b) Post-reconstruction: the forearm defect was reconstructed using a free anterolateral flap from the patient’s right thigh.

An 85-year-old woman presented to the Department of Dermatology with complaints of a subcutaneous mass on the dorsum of her left forearm that had grown rapidly over the course of the month prior. An excisional biopsy was performed on the day of her visit, yielding a pathological diagnosis of myxofibrosarcoma with positive margins. This diagnosis led to an immediate referral to the Department of Orthopaedic Surgery for a musculoskeletal oncology consultation. Based on the pathology results, the patient underwent an extended excision of her forearm, resulting in a 100 × 250 cm defect on the dorsal forearm (Fig. 1a). The defect was reconstructed 2 days later using a free anterolateral (ALT) flap harvested from the right thigh (Fig. 1b). The artery in the flap was anastomosed to the brachial artery via a side-to-end anastomosis and the vein was anastomosed to the accompanying brachial vein. The patient was transferred to the intensive care unit for 24 h of observation after the reconstructive surgery, and then to the orthopaedic ward. On the second day post-reconstruction, the patient was found in a state of cardiopulmonary arrest in her bed by a nurse who went in the room to clear her lunch plates. The patient had food residue in her mouth and around the pillow and was, therefore, suspected of asphyxiating due to food aspiration. CPR was initiated and return of spontaneous circulation (ROSC) was achieved after 10 min of CPR, during which 2 mg of adrenaline were administered.

The patient was readmitted to the intensive care unit; however, the total arrest time was estimated to be 20 min. Computed tomography (CT) of the head revealed severe hypoxic encephalopathy, and blood tests revealed a Neuron-Specific Enolase level of 288ng/ml, meaning that severe brain damage and poor neurological outcomes were expected.

**Fig. 2:**
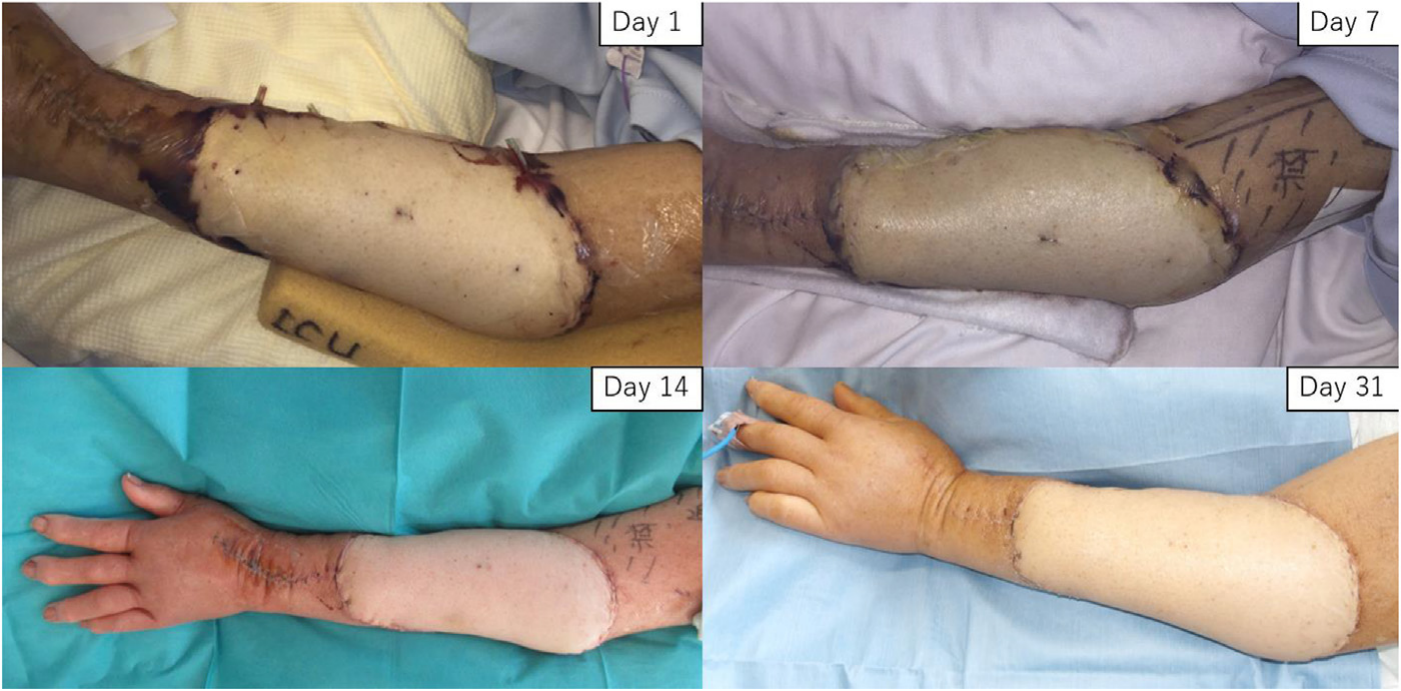
The appearance of the free flap on days 1, 7, 14, 31 post-cardiac arrest. We observed no signs of necrosis, vascular compromise, or infection in the free flap for ≥ 30 days post-cardiac arrest.

We closely observed the free flap and found no signs of necrosis, vascular compromise, or infection for ≥ 30 days post-arrest (Fig. 2). We also measured the regional oxygen saturation of the skin of the flap during the first 15 days and found no major changes. The regional oxygen saturation was measured at the following three points of the skin of the flap; The vascular pedicle, the distal portion of the flap, and the proximal portion of the flap. The measurements are shown in Table 1.

**Table 1:** Regional oxygen saturation of the skin of the flap.

POD (day)	1	3	4	5	7	8	10	11	15
Pedicle (%)	40	42	30	40	36	40	42	42	40
Proximal (%)	n.d.	34	25	n.d.	28	33	32	32	32
Distal (%)	n.d.	34	26	n.d.	30	30	32	30	35

*Abbreviations*: POD, post operative day; n.d.no data.

The patient was transferred to a nursing facility on post-operative day 53, with no recovery of consciousness or loss of cognitive function. At the time of her discharge, the patient’s free flap showed no signs of necrosis, vascular compromise, or infection.

## Literature review and discussion

We have reported herein the case of an 85-year-old woman who experienced cardiopulmonary arrest and was successfully resuscitated 2 days post-free flap transfer, who showed no compromise of the free flap for ≥ 30 days post-arrest.

The occurrence of post-operative cardiac arrest after free flap surgery is rare. Our department performed 144 free flap transfers between June 2017 and June 2024, during which time we encountered two other cases, for a total of three occurrences (incidence = 0.02%) of post-operative cardiac arrest after free tissue transfer between June 2017 and June 2024. This incidence was similar to that reported by Diaddigo et al.^1^

Known risk factors for catastrophic medical outcomes after microvascular free flap transfer, including cardiac arrest, include patient age, low body mass index, prolonged surgery time, and head and neck reconstructions.^1^ It is particularly challenging in head and neck reconstruction as a substantial number of patients with head and neck cancer suffer from cancer cachexia which worsens head and neck reconstruction free flap outcomes.^3^

In an ultra-aging society, an increasing number of elderly patients are becoming candidates for microsurgical reconstruction. Previous studies have shown that flap success rate or reoperation rate did not significantly decrease in elderly patients. However, postoperative systemic complications and mortality rates were significantly higher.^4^, ^5^, ^6^ Age alone should not be a contraindication for microsurgical reconstruction; instead careful risk stratification based on a meticulous assessment of comorbidities and physiological status should be conducted to determine whether the patient is eligible for reconstructive microsurgery.^4^ Pre-operative Frailty index scores or nutritional index scores may guide us in such decision making process.^7^^,^^8^

In the present case, the patient possessed good physical function and nutritional status with no frailty or serious comorbidities. After thorough discussion with the orthopedic oncology team and the patient, we decided to proceed with the reconstructive microsurgery. Postoperatively, measures were implemented to prevent physical deconditioning. Early mobilization was initiated and oral feeding was resumed on the second day after the surgery in accordance with Enhanced Recovery After Surgery (ERAS) pathways.^9^

The literature on the survival of free flaps after cardiac arrest is limited, as evidenced by a literature search of PubMed using the keywords “free flap” and “cardiac arrest” which returned three cases reporting the survival of free flaps after cardiac arrest and subsequent resuscitation.^10^, ^11^, ^12^ Combined with the three cases encountered in our department, we found six cases of seven free flaps that survived both cardiac arrest and resuscitation. We analyzed and compared the six cases, the findings of which are summarized in Table 2.

**Table 2:** Characteristics of free flaps that survived after cardiac arrest.

No	Age	Sex	Illness	Flap	POD	Arrest time	CPR time	Adrenaline dose	Noradrenaline dose
Current case	85	F	myxofibrosarcoma	free-ALT	2	20 mins	10 mins	2 mg 3 mg	
Case 2	68	M	tongue cancer	free-ALT	8	20 mins	-	2 mg -	
Case 3	46	F	tongue cancer	free-ALT	8	3 mins	46 mins	9 mg. 11 mg	
Pratt et al. (3)	38	F	breast cancer	free-DIEP	0	-	5 mins	2 mg -	
Young et al. (4)	54	M	tongue cancer	free-ALT	0	-	4 mins	n.d.	-
				free-fibular flap			-		
Purrinos et al. (5)	68	M	laryngeal cancer	free-forearm flap	0	-	15 mins	15 mins	-

*Abbreviations*: POD, post operative day; CPA, cardiopulmonary arrest; CPR, cardiopulmonary resuscitation; ALT, anterolateral thigh; DIEP, deep inferior epigastric perforator.

### Baseline characteristics

A total of three (50%) men and three (50%) women were included in this analysis, with a mean age of 59.3 (38–85) years. Three (50%) patients had tongue cancer and one (16.7%) each had breast cancer, laryngeal cancer, and malignant soft tissue tumor.

### Flap types

Four (66.7%) patients underwent reconstruction with a free ALT flap, and one (16.7%) with a free deep inferior epigastric perforator flap, one (16.7%) with a free radial forearm flap, one (16.7%) with a free fibular flap.

### Timing and cause of cardiopulmonary arrest

Three (50%) patients experienced intra-operative cardiac arrest, two (33.3%) experienced cardiac arrest on post-operative day (POD) 8, and one (16.7%) on POD 2. The mean time of duration of cardiac arrest was 7.2 (0–20) min. Two (33.3%) patients arrested due to airway obstruction, and one (16.7%) each due to pulmonary embolism, cardiac tamponade, and hypokalemia. The cause of arrest was not disclosed for one patient.

### CPR time and dose of drugs administered

The mean CPR duration was 16 (4–46) min and the mean dose of adrenaline administered was 4 (2–9) mg. Noradrenaline was administered in 2 patients after cardiac arrest in the intensive care unit. The amount of noradrenaline administered during the 48 h after cardiac arrest in the current case was 3 mg. In the other case, 11 mg of adrenaline was administered.

The mean cardiac arrest time among the six cases was 7.2 min with a maximum duration of 20 min. In a pig model of cardiac arrest, carotid blood flow fell to zero in 4 min and coronary blood flow fell to zero in 1 min.^13^ From this, it can be hypothesised that blood flow to the free flap stops within a few minutes of cardiac arrest. In contrast, blood flow to the flap is clamped during free tissue transfer, creating intra-operative ischaemia. The correlation between the intra-operative ischaemic time and post-operative complications has been a topic of constant discussion. Politano et al.^14^ conducted a meta-analysis of 14 studies and found that ischaemia time was not associated with acute post-operative flap complications, and suggested that the outcomes of free flap transfer were not affected when the ischaemia time was < 4 h.^14^ In case of free jejunal transfer, a free flap susceptible to ischemia, Kagaya et al.^15^ found that intraoperative ischemia time for free jejunal transfer was not correlated to the risk of postoperative complications.^15^ Therefore, although perfusion to the free flap declined to nearly zero within a few minutes after cardiac arrest, such a loss of blood supply to the flap may not negatively affect its survival.

The traditional thought among microsurgeons is that the use of vasopressors, including adrenaline, causes vasospasm and leads to flap failure; therefore, the use of vasopressors should be avoided.^16^^,^^17^ A survey by Marsh et al.^17^ among microsurgeons who perform head and neck reconstruction in the UK found that 77% of the surgeons never use vasopressors post operatively.^17^^,^^18^

The same reluctance of the use of vasopressor among microvascular surgeons was found in a survey by the American society of Plastic surgeons and American Society of Reconstructive Microsurgeons.^16^^,^^18^ Such beliefs are not without reason. The use of vasopressor in septic shock patients have been known to result in irreversible ischemia and distal necrosis of the upper and lower extremities due to severe vasospasm.^19^^,^^20^ Patients who received α-adrenergic activating vasopressors were found to be more likely to sustain limb ischemia.^21^ These studies reflects the common belief among microsurgeons that the use of vasopressors are harmful for free flaps survival and that the avoidance of vasopressors in free flap surgery is a widespread professional opinion.

However, in recent years, multiple studies have shown that the use of intra-operative vasopressors is not associated with flap-related post-operative complications. Michelle et al.^22^ conducted a systematic review and meta-analysis to investigate the outcomes of head and neck free flaps and vasopressor use during the intra-operative period. This study included 18 qualitative analyses and 9 meta-analyses from which the authors found no difference in flap failure rates with the use of intra-operative vasopressors during head and neck surgeries. In fact, the authors found that peri-operative vasopressor use decreased flap complications. Noori et al.^23^ also conducted a systematic review and meta-analysis that included 15 studies and 8427 flaps to investigate the harmful or beneficial effects of vasopressor use during free flap transfer. The authors found no evidence that vasopressor use increased adverse events. They actually found that vasopressor use reduced the risk of free flap failure. Studies have shown that the intra-operative use of vasopressors with no negative impact on free flap transfer is increasing. The vasopressors used in these studies included phenylephrine, ephedrine, norepinephrine and calcium chloride; however, no previous studies have investigated the use of adrenaline.

Adrenaline is a catecholamine that works as an inotrope, chronotrope, and vasopressor by directly or indirectly stimulating α1, α2, β1, and β2 receptors.^24^^,^^25^ Adrenaline is not commonly used in surgery for healthy individuals, and its use is often limited to a first-line treatment for severe conditions such as anaphylaxis and cardiac arrest.^25^^,^^26^ The purpose of the administering adrenaline during cardiac arrest is to increase aortic diastolic pressure which increases coronary and cerebral perfusion pressure by activating the alpha receptors on vascular smooth cells, thereby increasing the likelihood of ROSC.^27^^,^^28^ Adverse effects of adrenaline use include palpitations, headache, ventricular arrythmias, angina, and myocardial infarction.^29^ Some of which is attributed to the potent vasoconstrictive property of adrenaline. It is know that intravenous overdose cause coronary vasospasm in health adults and that such effect can even occur from local infiltration of therapeutic dose of adrenaline used to reduce hemorrhage.^29^ Literatures have also reported adverse neurological events associated with intramuscular administration of adrenaline including ischemic stroke and transient ischemia attack. It was suggested that such neurological events resulted from acute severe hypertension which was caused by the vasoconstriction mediated by the potent alpha adrenergic properties of adrenaline.^30^

We would hypothesized that the use of such a potent vasopressor would have negative consequences to free flaps. In our analysis, all six cases were administered adrenaline for resuscitation. The mean dose of adrenaline was 4 mg, and the maximum dose was 9 mg, with no flap failure. According to an analysis of 348,996 cases of in-hospital arrest in the United States, the mean dose of adrenaline in patients with ROSC was also 4 mg, indicating no difference in the mean dose administered. These findings were surprising, as we assumed that the use of adrenaline would constrict the vascular pedicle and lead to free flap failure. There are few literatures that studied the relationship between adrenaline administration and flap survival. We identified a study by Eley et al.^31^ which used laser Doppler velocimetry to study the skin blood flow in free flaps after the administration of epinephrine, norepinephrine, dobutamine, and dopamine. The authors found that the administration of epinephrine was associated with decreased blood flow to the skin of free flaps.^31^ In another study, although no significant statistical difference was observed, Krammer et al.^32^ found that the intraperitoneal injection of adrenaline (0.1mg/kg) tended to decrease skin flap survival in rats. However, we could not find any literature that studied the outcomes of intra-operative adrenaline use in free tissue transfer in human. To the best of our knowledge, ours is the first study to report the mean dose of adrenaline used for CPR in patients with free flaps that survived cardiac arrest. Consequently, our analysis adds to the evidence that cardiac arrest and subsequent CPR, including the administration of adrenaline, do not significantly affect the survival of free flaps.

Given that this study stemmed from a case report, there are a few limitations worth mentioning. First, the sample size was small. Second, no literature was included in which the free flap failed after cardiac arrest. Therefore, research involving multiple institutions is needed to further investigate the survivability of free flaps after cardiac arrest.

## Conclusion

Post-free flap transfer surgery cardiac arrest and subsequent cardiopulmonary resuscitation did not appear to significantly decrease free flap survival.

## Statement of informed consent

The authors have obtained written consent from the guardian of the patient for publication.

## Statement of human and animal rights

None.

## Funding

None.

## Data statement

None.

## Declaration of competing interest

None.
